# Antitumor activity of gambogic acid on NCI-H1993 xenografts via MET signaling pathway downregulation

**DOI:** 10.3892/ol.2021.12477

**Published:** 2021-01-20

**Authors:** Donglei Li, Huiwei Yang, Runpu Li, Yanli Wang, Weijun Wang, Dongjie Li, Shaolin Ma, Xuyu Zhang

Oncol Lett 10: 2802-2806, 2015; DOI: 10.3892/ol.2015.3719

Subsequently to the publication of the above paper, an interested reader drew to the authors’ attention that the panels shown in Fig. 3A showed some unexpected similarities when comparing the different panels.

The authors have re-examined their data and realized that Fig. 3A was assembled incorrectly as certain of the data panels had been mislabelled; however, the authors were able to reassemble this Figure based on the results they obtained from one of their repeated experiments, and the revised version of Fig. 3 is shown below. The authors regret the errors that were made during the preparation of the Figure, although they were able to confirm that these errors did not seriously affect the conclusions reported in the paper. The authors are grateful to the editor of *Oncology Letters* for allowing them the opportunity to publish a Corrigendum, and all the authors agree to this Corrigendum. Furthermore, they apologize to the readership for any inconvenience caused.

## Figures and Tables

**Figure 3.**
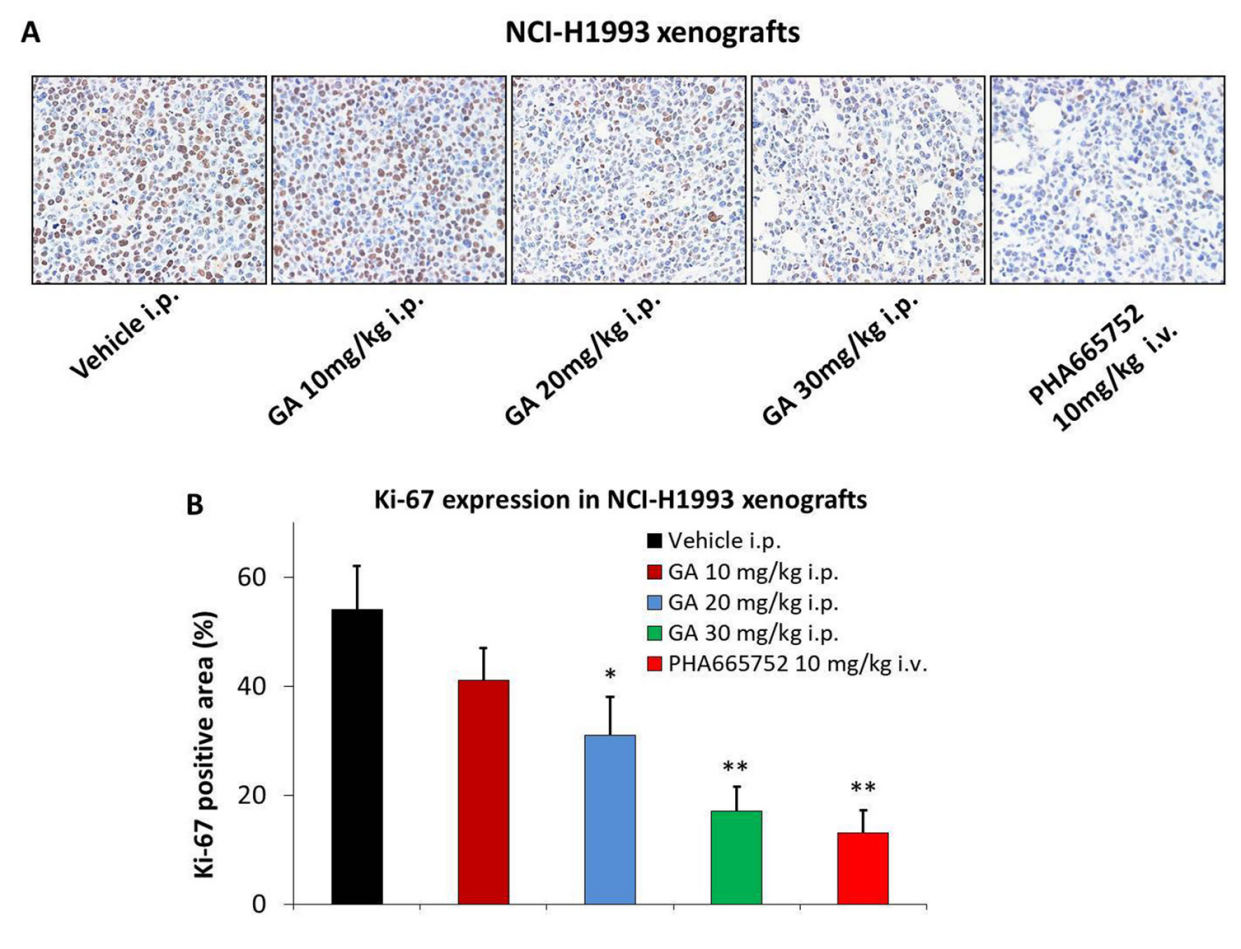
GA inhibits the expression of Ki-67 in the NCI-H1993 xenograft model. The NCI-H1993 xenograft model was treated with various doses of GA, vehicle or positive control for 2 h on day 21 of the efficacy study. (A) Representative images of Ki-67 staining. (B) Quantification of Ki-67 positive area (%). Values are expressed as the mean ± standard deviation, n=10. *P<0.05 vs. vehicle group; **P<0.01 vs. vehicle group. GA, gambogic acid; i.p., intraperitoneal; i.v., intravenous.

